# Interfacial Thermal Transport and Phonon Scattering of Graphene and Graphene Oxide Embedded in Calcium Silicate Hydrate: A Molecular Dynamics Study

**DOI:** 10.3390/nano16171105

**Published:** 2026-09-02

**Authors:** Tong Chen, Dan Chen, Cheng Gong, Yongzhe Zhao, Yongliang Han, Yijie Wang

**Affiliations:** 1 Shaanxi Coal Industry Fengjing New Energy Technology Co., Ltd., Xi’an 710300, China; 2 CCTEG Xi’an Geothermal Energy Development Co., Ltd., Xi’an 712100, China; 3 CCTEG Xi’an Research Institute (Group) Co., Ltd., Xi’an 710077, China

**Keywords:** graphene oxide, calcium silicate hydrate, thermal conductivity, interfacial binding, radial distribution function, phonon scattering, molecular dynamics

## Abstract

Graphene and graphene oxide (GO) are promising nanofillers for improving thermal transport in cementitious materials, but their performance is strongly affected by interactions with calcium silicate hydrate (C-S-H). Understanding how these fillers retain or lose their heat-transport capability after incorporation into the cement hydrate matrix is therefore important for rational nanocomposite design. Reverse non-equilibrium molecular dynamics simulations were conducted to compare isolated graphene/GO sheets with the corresponding sheets embedded in C-S-H. The extrapolated thermal conductivity of pristine graphene decreased from 1854.6 to 1264.2 W/(m·K) after embedding, giving a retention ratio of 0.68. Increasing the oxidation degree reduced the intrinsic conductivity of GO through defect-induced phonon scattering, while the additional reduction caused by C-S-H progressively weakened. At an oxidation degree of 20%, GO retained more than 90% of its isolated-sheet conductivity. Atomic heat-flux analysis showed that C-S-H markedly broadened the transport-direction distribution of graphene but produced only limited additional disturbance in GO. Interfacial binding energy increased with oxidation degree, and radial distribution function analysis identified short-range Ca-O coordination and hydrogen bonding at the GO/C-S-H interface. Phonon density of states analysis further revealed pronounced substrate-induced phonon softening in graphene, whereas the vibrational spectrum of GO remained comparatively stable. These results clarify the trade-off between intrinsic conductivity and matrix-induced thermal stability in graphene-based cementitious nanocomposites.

## 1. Introduction

Cement concrete is one of the most widely used construction materials. Considerable heat is released during cement hydration, whereas the relatively low thermal conductivity of the cement matrix limits internal heat dissipation [1,2,3]. In massive concrete structures, the difference between the internal and surface temperatures can produce tensile stresses and increase the risk of early-age thermal cracking. Conventional measures, including cooling pipes and surface insulation, can reduce temperature rise at the structural scale, but modification of the intrinsic thermal transport properties of cementitious materials provides an additional approach for improving thermal management. Recent studies further show that the performance of cement-based construction materials can be modified through solid-waste-assisted binder design, while material composition strongly affects cracking potential and viscoelastic response [4,5].

Carbon-based nanomaterials have attracted extensive attention because of their high aspect ratio and excellent thermal and mechanical properties [6,7,8]. Graphene exhibits an intrinsic in-plane thermal conductivity of several thousand W/(m·K) and is therefore considered a potential thermally conductive filler. However, the thermal conductivity enhancement obtained in a composite is usually far below the value predicted from the intrinsic conductivity of the filler [9,10]. A major reason is the substrate effect. The mismatch in vibrational spectra and acoustic impedance between graphene and the surrounding matrix introduces interfacial phonon scattering and thermal resistance, thereby reducing the effective heat-transfer capability of the filler [11]. Graphene oxide differs from pristine graphene because hydroxyl and epoxy groups are distributed on the basal plane, while carboxyl and carbonyl groups may occur near the sheet edges [12,13]. These oxygen-containing functional groups disrupt the sp^2^ carbon network and reduce the intrinsic thermal conductivity of GO [14]. At the same time, they improve water dispersibility and strengthen interactions with inorganic matrices. In cementitious systems, oxygen atoms in GO can interact with calcium ions and interlayer water in C-S-H, producing Ca-O coordination, hydrogen bonding, and electrostatic attraction [15,16]. These interactions may change the interfacial phonon coupling and the additional thermal resistance introduced by the matrix.

C-S-H is the principal hydration product of Portland cement and accounts for approximately 50–70% of the solid hydration products. Its layered and chemically heterogeneous structure makes it the primary nanoscale substrate for graphene-based fillers in the cement matrix [17,18]. Previous molecular dynamics studies have investigated the mechanical behavior, interfacial sliding, and chemical interaction of graphene or GO with C-S-H [15,16,17,18]. Studies on the thermal behavior of graphene-based composites have also shown that substrate-induced phonon scattering can strongly suppress the conductivity of the filler [9,10,11]. Nevertheless, the relationship between GO oxidation degree, C-S-H interfacial interaction, heat-flux redistribution, and thermal-conductivity retention remains insufficiently clarified. Recent studies further demonstrate the relevance of GO/C-S-H architectures to thermal-energy-related cementitious applications [19], while broader analyses of graphene phonon transport emphasize the strong dependence of heat conduction on phonon spectrum, defects, substrate coupling, and model length [20]. These observations motivate a direct comparison between intrinsic oxidation-induced scattering and the additional perturbation imposed by C-S-H. Reverse non-equilibrium molecular dynamics (RNEMD) simulations were employed to investigate the thermal transport behavior of isolated and C-S-H-embedded graphene/GO sheets with oxidation degrees ranging from 0% to 20%. Size-dependent thermal conductivity, conductivity-retention ratio, atomic heat-flux vectors, interfacial binding energy, radial distribution functions, and phonon density of states were analyzed to clarify the distinct responses of pristine graphene and oxidized GO to the C-S-H substrate. The analyses establish an atomistic mechanism linking oxidation-induced disorder, interfacial interactions, and substrate-induced phonon scattering in graphene-based cementitious composites.

Unlike previous studies that mainly focused on mechanical behavior, interfacial sliding, and chemical interactions, this study links oxidation-dependent thermal-conductivity retention with atomic heat-flux redistribution and phonon-spectrum evolution in graphene/GO–C–S–H systems.

## 2. Modeling and Simulation Methods

### 2.1. Construction of the C-S-H and Graphene/GO Models

The atomistic model of C-S-H was constructed from an 11 Å tobermorite structure following the realistic molecular model proposed for cement hydrates [17,18]. Selected silicate bridging units were randomly removed to reproduce the defective chain structure of C-S-H. The resulting *Q^n^* distribution was *Q*^0^ = 11.6%, *Q*^1^ = 65.1%, and *Q*^2^ = 23.3%, and the calcium-to-silicon ratio was approximately 1.67. Water molecules were introduced into the interlayer space using the grand canonical Monte Carlo method until a water density of approximately 1.0 g/cm^3^ was reached [21,22].

**Figure 1:**
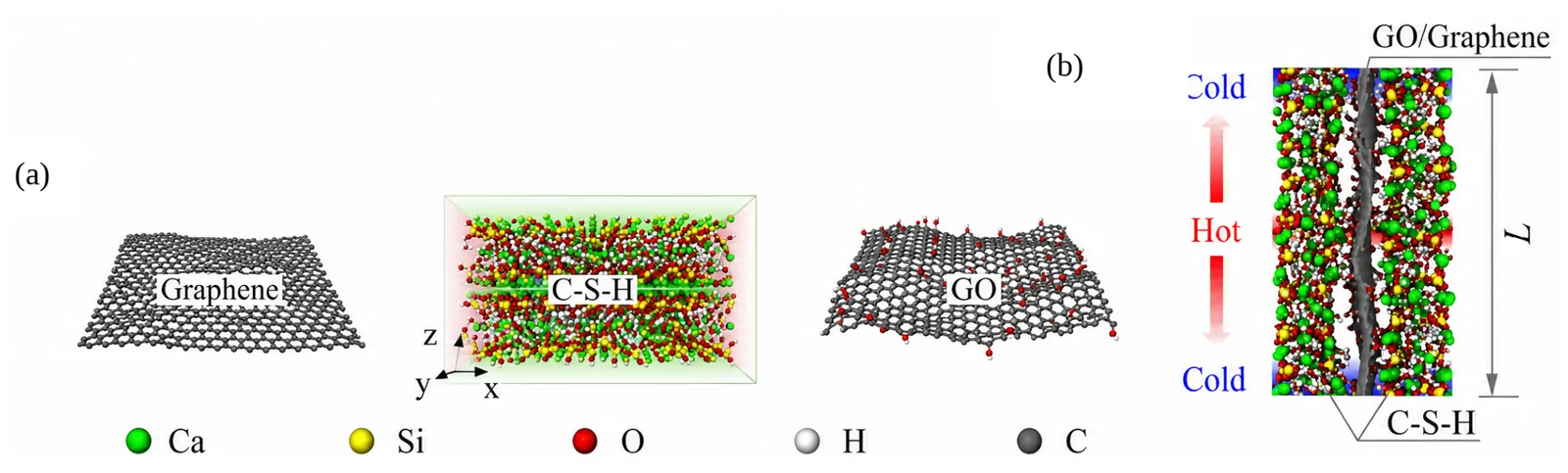
(**a**) Atomistic structures of graphene, C-S-H, and graphene oxide; (**b**) graphene/GO embedded in C-S-H and the arrangement of the hot and cold regions.

Graphene and GO sheets were placed in the central region of the expanded C-S-H cell. The transverse size of the sheet was approximately 2.45 nm and was matched to the C-S-H model. Hydroxyl and epoxy groups were randomly distributed on both sides of the GO basal plane at a ratio of 1:1. The oxidation degree, *R*, was defined as the proportion of oxidized carbon sites and was varied from 0% to 20%. *R* = 0% represents pristine graphene. Models with different lengths along the heat-transfer direction were established to evaluate the size dependence of thermal conductivity. For the interfacial analysis, graphene/GO sheets were placed in contact with the yz-, xz-, and xy-planes of the C-S-H model. The constructed atomistic models and the RNEMD hot/cold-region arrangement are shown in Figure 1.

### 2.2. Simulation Procedure and Force Field

All molecular dynamics simulations were performed using the 2021 version of the LAMMPS package (https://www.lammps.org/; accessed on 31 August 2026) [21]. The ReaxFF reactive force field was adopted to describe interactions among Ca, Si, O, H, and C atoms because it accounts for bond formation, bond breaking, electrostatic interactions, and changes in the local chemical environment. The parameterization developed for hydrated calcium-silicate systems and related interfaces was adopted following Refs. [23,24,25]. The initial structures were optimized using the conjugate gradient algorithm with an energy convergence criterion of 1.0 × 10^−4^ kcal/mol.

A unified ReaxFF framework was retained for both isolated and embedded systems to maintain a consistent description of Ca-Si-O-H-C interactions across C-S-H, graphene/GO, and their interfaces. This choice is particularly relevant to oxygen-containing functional groups, interfacial coordination, and bond-order changes in the chemically heterogeneous GO/C-S-H system. Yang and Cao previously applied ReaxFF to graphene/GO-C-S-H interfacial heat-transfer simulations, using a C-H-O-Ca-Si reactive description and phonon-based analysis to investigate interfacial thermal transport [26]. Diao et al. further applied ReaxFF to the thermal transport of graphene-based carbon systems [27], while systematic comparisons by Si et al. among Tersoff, REBO, optimized Tersoff, and AIREBO potentials demonstrated that the absolute thermal conductivity of graphene can depend strongly on the selected interatomic potential [28]. Accordingly, the present study emphasizes relative changes between identically parameterized isolated and embedded systems and treats the absolute graphene conductivity as a model-dependent reference.

After geometry optimization, the systems were equilibrated in the NPT ensemble at 300 K and 1 atm for 0.5 ns using Nosé–Hoover temperature and pressure control. The equilibrated configurations were then relaxed in the NVE ensemble for 0.1 ns before thermal transport calculations. A time step of 0.25 fs was applied. To relax the random distribution of oxygen-containing groups, the GO system was heated from 300 to 500 K, maintained at 500 K, cooled to 300 K, and equilibrated at 300 K. Periodic boundary conditions were applied in all directions. The main model and simulation parameters are summarized in Table 1.

### 2.3. Thermal Conductivity Calculation

**Figure 2:**
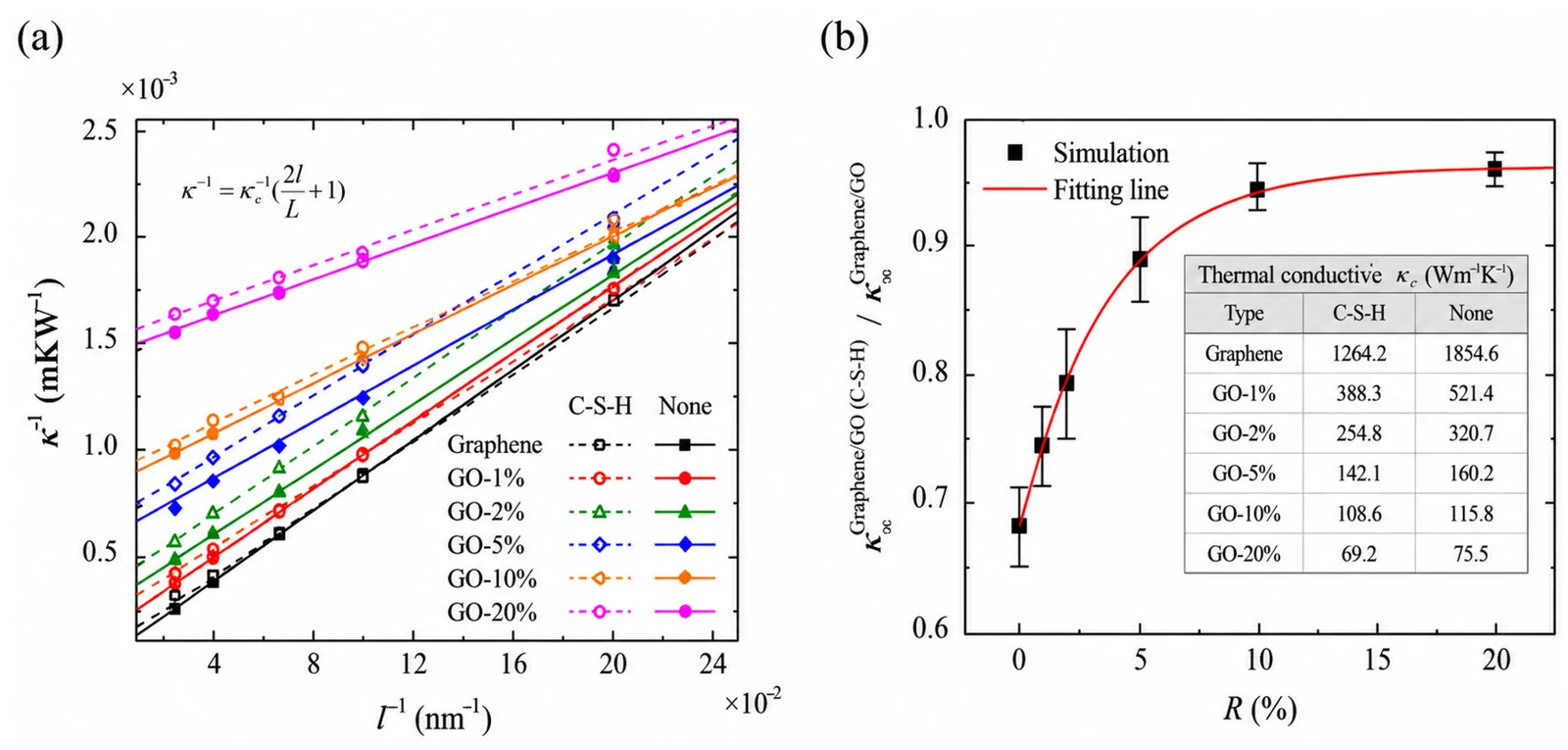
(**a**) Relationship between inverse thermal conductivity and inverse model length; (**b**) thermal conductivity and retention ratio of isolated and C-S-H-embedded graphene/GO as functions of oxidation degree. Error bars in panel (**b**) represent the standard deviation of five block-averaged thermal conductivity values obtained from the final 1.0 ns production trajectory (*n* = 5).

The thermal conductivity was calculated using the RNEMD method proposed by Müller-Plathe [29]. The simulation cell was divided into slabs along the heat-transfer direction. A hot region was located in the center of the cell, while cold regions were placed at both ends. Kinetic energy was exchanged between selected atoms every 1000 time steps, thereby producing two symmetric heat-flow paths. The imposed heat flux was calculated as

(1) $$J=\frac{1}{2t}\sum m\left(v_{h}^{2}-v_{c}^{2}\right)$$

where *J* is the imposed heat-flux rate generated by the kinetic-energy exchanges, *m* is the atomic mass, *v_h_* and *v_c_* are the velocities of the selected atoms in the hot and cold slabs, respectively, and *t* is the accumulated exchange time. The factor 1/2 accounts for the two symmetric heat-flow paths. After a stable non-equilibrium temperature distribution was established, the thermal conductivity was obtained from Fourier’s law:

(2) $$\kappa =\frac{J}{2A\left|\partial T/\partial z\right|}$$

where *κ* is the thermal conductivity, *A* is the cross-sectional area perpendicular to the heat-flow direction, and *∂T/∂z* is the fitted temperature gradient. The factor 2 in Fourier’s law accounts for the two symmetric heat-flow paths. For each steady-state temperature profile, the fitting interval was selected from the approximately linear region. The nonlinear temperature regions adjacent to the hot and cold exchange slabs were excluded rather than removing a fixed number of slabs in every simulation. The remaining linear portion was fitted by least squares to determine the temperature gradient *∂T/∂z*. The first 2.0 ns of the RNEMD simulation were used to establish the steady state, and the final 1.0 ns was used for data collection. The production trajectory was divided into five equal time blocks (*n* = 5), and the error bars represent the standard deviation of the five block-averaged thermal conductivities. The effective infinite-length conductivity was then estimated by fitting inverse conductivity against inverse model length:

(3) $$\frac{1}{\kappa_{L}}=\frac{1}{\kappa_{\infty }}\left(1+\frac{2l}{L}\right)$$

where *κ_L_* is the conductivity of a model with length *L*, *κ*_∞_ is the extrapolated infinite-length conductivity, and *l* is the effective phonon mean free path. Equation (3) was used as a finite-size extrapolation based on the set of model lengths shown in Figure 2a. The shorter models were retained because the finite-size effect is more pronounced at small *L* and these data provide an important part of the variation in 1/*L* used to characterize the size dependence. Because phonon mean free paths in graphene can be comparable to or larger than the present nanoscale model dimensions, *κ*_∞_ is interpreted as an effective extrapolated reference within the present ReaxFF/RNEMD framework rather than a unique bulk intrinsic constant [20,30]. The thermal-conductivity–retention ratio was defined as *κ*_embedded_/*κ*_isolated_.

### 2.4. Interfacial Binding, RDF, and Vibrational Analysis

The interfacial binding energy was calculated to evaluate the stability of the graphene/GO-C-S-H systems:

(4) $$E_{bind}=E_{C-S-H}+E_{filler}-E_{total}$$

where *E*_total_ is the potential energy of the composite, *E*_C-S-H_ is the potential energy of the isolated C-S-H substrate, *E*_filler_ is the potential energy of the isolated graphene or GO sheet in the same configuration, and *E*_bind_ is the interfacial binding energy obtained from Equation (4). A larger positive *E*_bind_ represents stronger interfacial attraction.

The RDF, *g*(*r*), was calculated to characterize the local atomic structure at the GO/C-S-H interface. The Ca-O pair between calcium atoms in C-S-H and oxygen atoms in GO was analyzed to identify calcium–oxygen coordination, while the H-O pair was analyzed to identify hydrogen bonding between interlayer water or surface hydroxyl groups and GO oxygen atoms. RDF curves were computed from the production trajectory using the same sampling interval and statistical window for all oxidation degrees. Here, *r* is the pair separation and *g*(*r*) is the local pair density normalized by the corresponding average pair density.

Atomic heat-flux vectors were extracted to visualize the spatial direction of energy transport. The vector contribution of atom *i* was obtained from its energy, velocity, and stress tensor. The magnitude and angular distributions were compared for isolated and embedded fillers. The PDOS was calculated from the Fourier transform of the velocity autocorrelation function:

(5) $$P\left(\omega \right)=\frac{1}{\sqrt{2\pi }}\int_{0}^{t_{max}}e^{i\omega t}\left[\sum_{j}v_{j}\left(t\right)\cdot v_{j}\left(0\right)\right]dt$$

where *P*(*ω*) is the spectral intensity at angular frequency *ω*, *i* is the imaginary unit, *t*_max_ is the maximum correlation time, v*_j_*(*t*) is the velocity vector of atom *j* at time *t*, and v*_j_*(0) is its initial velocity vector. The summation is performed over the analyzed atoms, and the dot denotes the vector scalar product. Total and out-of-plane components were analyzed to identify changes in vibrational modes caused by the C-S-H substrate.

## 3. Results and Discussion

### 3.1. Size Effect and Thermal-Conductivity Retention

The thermal conductivity of graphene and GO exhibits a clear length dependence. The calculated conductivity increases with model length and gradually approaches a stable value over the simulated range. The inverse conductivity shows an approximately linear relationship with inverse length, supporting the use of Equation (3) as an effective finite-size correction within the investigated model-length window. The approximately linear inverse-conductivity trend agrees with the phonon mean-free-path description commonly applied to graphene-based systems [31,32,33], although length-dependent thermal transport in graphene can persist over substantially larger length scales [20,30]. For broader methodological context on nanoscale energy transport, defect effects, and strain-sensitive thermal response in low-dimensional materials, see Refs. [34,35,36].

The extrapolated thermal conductivity of isolated pristine graphene is 1854.6 W/(m·K). After embedding in C-S-H, the value decreases to 1264.2 W/(m·K) and the retention ratio is 0.68. The marked decrease indicates that the C-S-H substrate introduces additional scattering into the highly ordered graphene lattice. The heterogeneous atomic structure, interlayer water, and local electrostatic environment of C-S-H disturb the flexural and in-plane vibrational modes of graphene, thereby reducing the effective phonon mean free path.

The value of 1854.6 W/(m·K) is therefore treated as a ReaxFF-based extrapolated reference rather than a universal intrinsic thermal conductivity. Force-field comparisons have shown that graphene thermal conductivity and phonon behavior are sensitive to the adopted interatomic potential [27,28]. Using the same ReaxFF description and the same size-correction procedure for both isolated and embedded systems limits inconsistency in the paired comparison; therefore, the thermal-conductivity–retention ratio is considered more robust for interpreting the substrate effect than the absolute conductivity alone.

Increasing the oxidation degree causes a rapid decrease in the intrinsic thermal conductivity of the isolated GO sheet. Oxygen-containing functional groups change the local mass distribution, disrupt the sp^2^ carbon network, and generate structural distortion, all of which enhance intrinsic phonon scattering [14]. A similar decreasing trend is observed after GO is embedded in C-S-H. However, the difference between the isolated and embedded states becomes progressively smaller as *R* increases. At *R* = 20%, the embedded GO sheet retains more than 90% of the conductivity of the isolated sheet. The retention ratio approaches a plateau when the oxidation degree reaches approximately 10%.

The oxidation-dependent conductivity-retention behavior reflects two competing effects. First, functional groups reduce the absolute thermal conductivity of GO by introducing defects. Second, the intrinsic disorder generated by these defects weakens the relative contribution of additional matrix-induced scattering. Therefore, highly oxidized GO does not have a higher absolute conductivity than graphene, but its conductivity is less sensitive to the transition from an isolated state to a C-S-H-embedded state.

### 3.2. Atomic Heat-Flux Redistribution

The atomic heat-flux vectors of isolated graphene are predominantly aligned with the imposed heat-flow direction. The spatial vector map shows a narrow and continuous in-plane transport path, and the angular distribution is concentrated near 0°. The concentrated in-plane heat-flux distribution is consistent with the long phonon mean free path and high thermal conductivity of the ordered carbon lattice.

**Figure 3:**
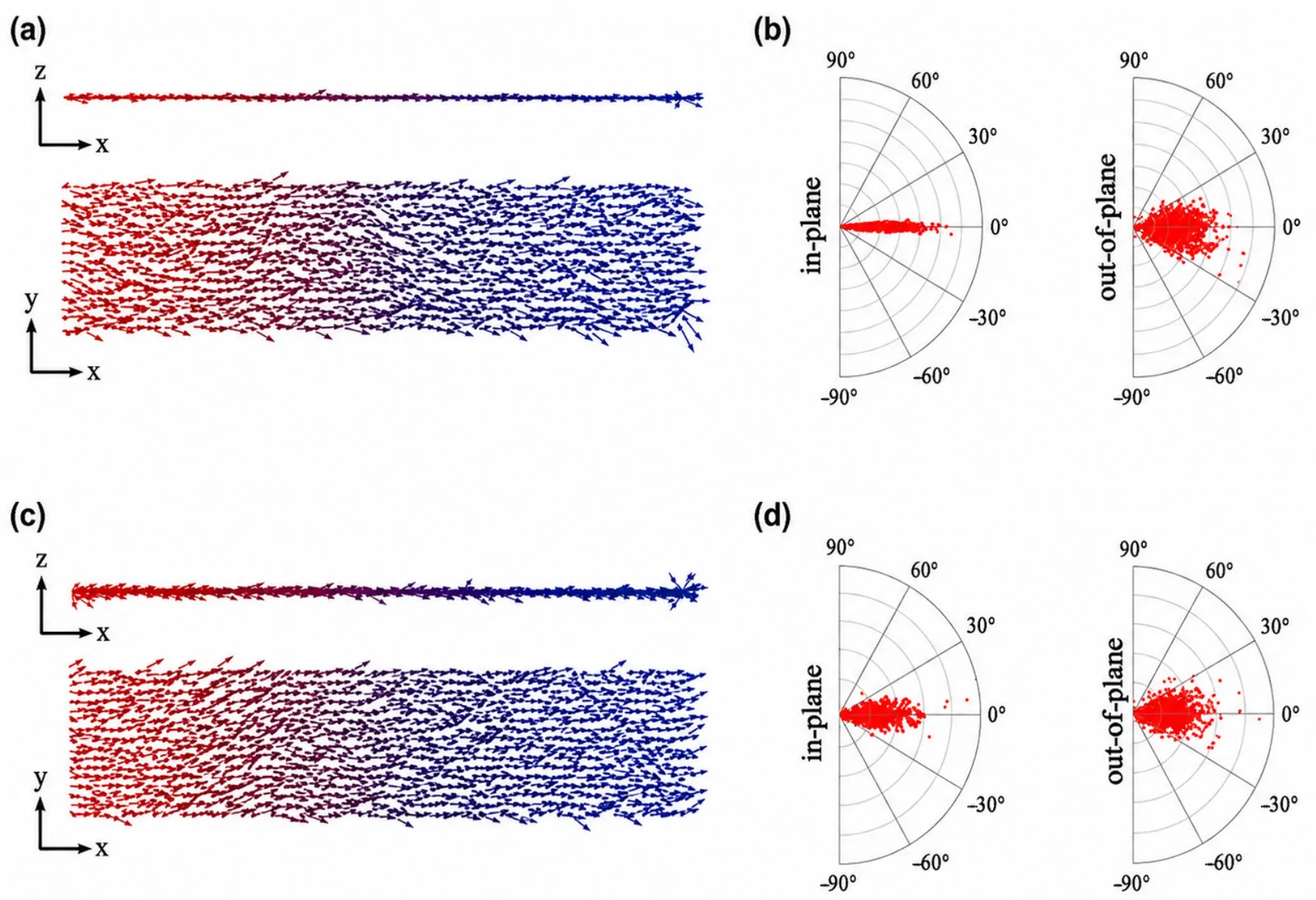
Spatial distribution, magnitude, and angular distribution of atomic heat-flux vectors for isolated graphene and graphene embedded in C-S-H. Panels: (**a**) spatial heat-flux vector distribution of isolated graphene; (**b**) in-plane and out-of-plane angular distributions of isolated graphene; (**c**) spatial heat-flux vector distribution of graphene embedded in C-S-H; (**d**) corresponding in-plane and out-of-plane angular distributions of embedded graphene. The red-to-blue color gradient indicates a decrease in relative local thermal-energy level from the hot region to the cold region, and the arrows indicate the heat-transfer direction.

After graphene is embedded in C-S-H, the out-of-plane component increases substantially and the angular distribution broadens to approximately −30° to 30°. The broader distribution indicates that the substrate redirects part of the originally aligned heat flux. Interactions with calcium, silicate groups, and interlayer water disturb the flexural modes of graphene and increase the probability of directional scattering. The vector analysis therefore provides a direct explanation for the reduction in thermal conductivity and the low retention ratio of pristine graphene. The corresponding spatial and angular heat-flux distributions are shown in Figure 3.

The heat-flux vectors of GO at *R* = 10% are more dispersed than those of pristine graphene even in the isolated state. Local distortions and oxygen-containing groups cause the transport direction to deviate from the graphene basal plane. After the GO sheet is embedded in C-S-H, the vector field and the angular distribution change only slightly. The C-S-H matrix therefore introduces less additional disturbance than it does in pristine graphene.

**Figure 4:**
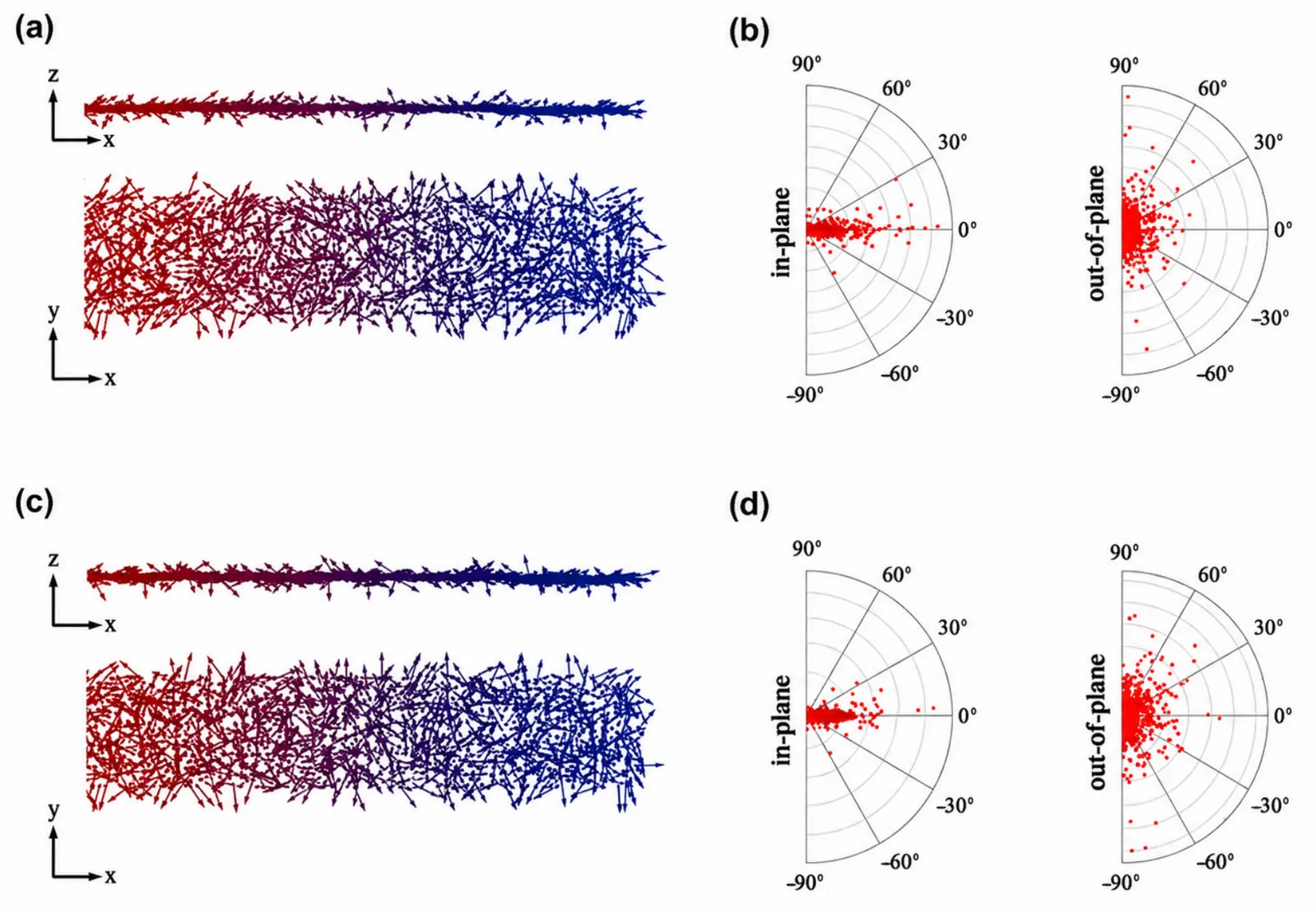
Spatial distribution, magnitude, and angular distribution of atomic heat-flux vectors for isolated GO at *R* = 10% and GO embedded in C-S-H. Panels: (**a**) spatial heat-flux vector distribution of isolated GO at R = 10%; (**b**) in-plane and out-of-plane angular distributions of isolated GO; (**c**) spatial heat-flux vector distribution of GO embedded in C-S-H; (**d**) corresponding in-plane and out-of-plane angular distributions of embedded GO. The red-to-blue color gradient indicates a decrease in relative local thermal-energy level from the hot region to the cold region, and the arrows indicate the heat-transfer direction.

The limited matrix-induced change indicates that oxidation-induced scattering dominates the thermal transport behavior of GO. Once the intrinsic disorder becomes sufficiently strong, the surrounding C-S-H contributes only a relatively small additional perturbation. The limited matrix-induced change is consistent with the gradual increase and eventual plateau of the thermal-conductivity–retention ratio. The corresponding heat-flux distributions for GO are shown in Figure 4.

### 3.3. Interfacial Binding Energy and RDF Analysis

The interfacial binding energy increases monotonically with the oxidation degree of GO. Pristine graphene interacts with C-S-H mainly through van der Waals attraction, whereas oxygen-containing groups provide additional electrostatic and coordination interactions. Consequently, GO shows stronger interfacial adhesion than graphene. Among the investigated contact planes, the xy-plane of C-S-H exhibits the highest binding energy, followed by the yz- and xz-planes. The difference is related to the layered C-S-H structure and the surface exposure of calcium ions and interlayer water.

**Figure 5:**
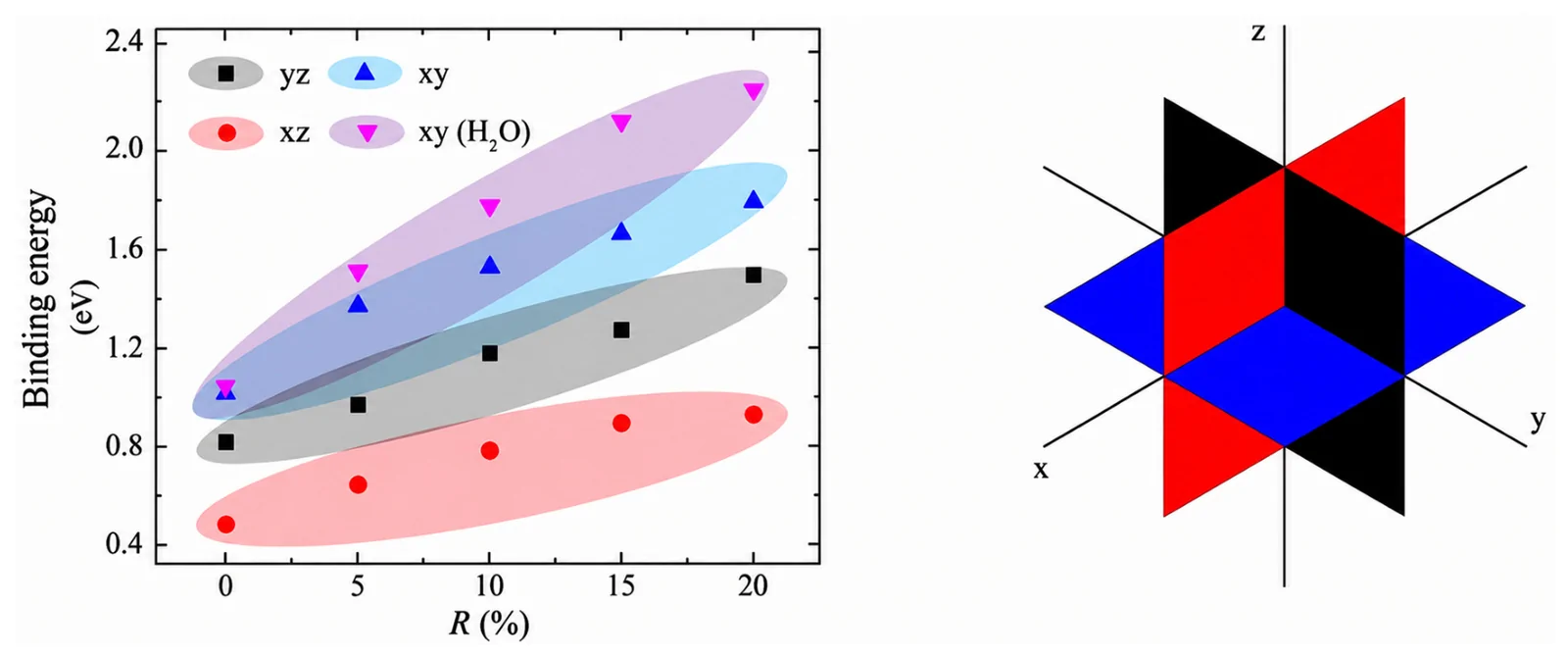
Interfacial binding energies between C-S-H and graphene/GO sheets with different oxidation degrees and contact planes. In the binding-energy plot, black squares, red circles, blue upward triangles, and purple downward triangles denote the yz, xz, xy, and xy (H_2_O) configurations, respectively; the shaded regions use the corresponding colors.

The xy-plane provides a relatively high density of accessible calcium sites. Oxygen atoms in hydroxyl and epoxy groups can coordinate with these calcium ions, forming Ca-O bridges at the interface. Water molecules located near the C-S-H surface can also form hydrogen bonds with GO functional groups. The combined effect of calcium bridging, hydrogen bonding, and electrostatic attraction stabilizes the GO/C-S-H interface and improves vibrational coupling between the two components. The oxidation- and contact-plane-dependent binding energies are summarized in Figure 5.

**Figure 6:**
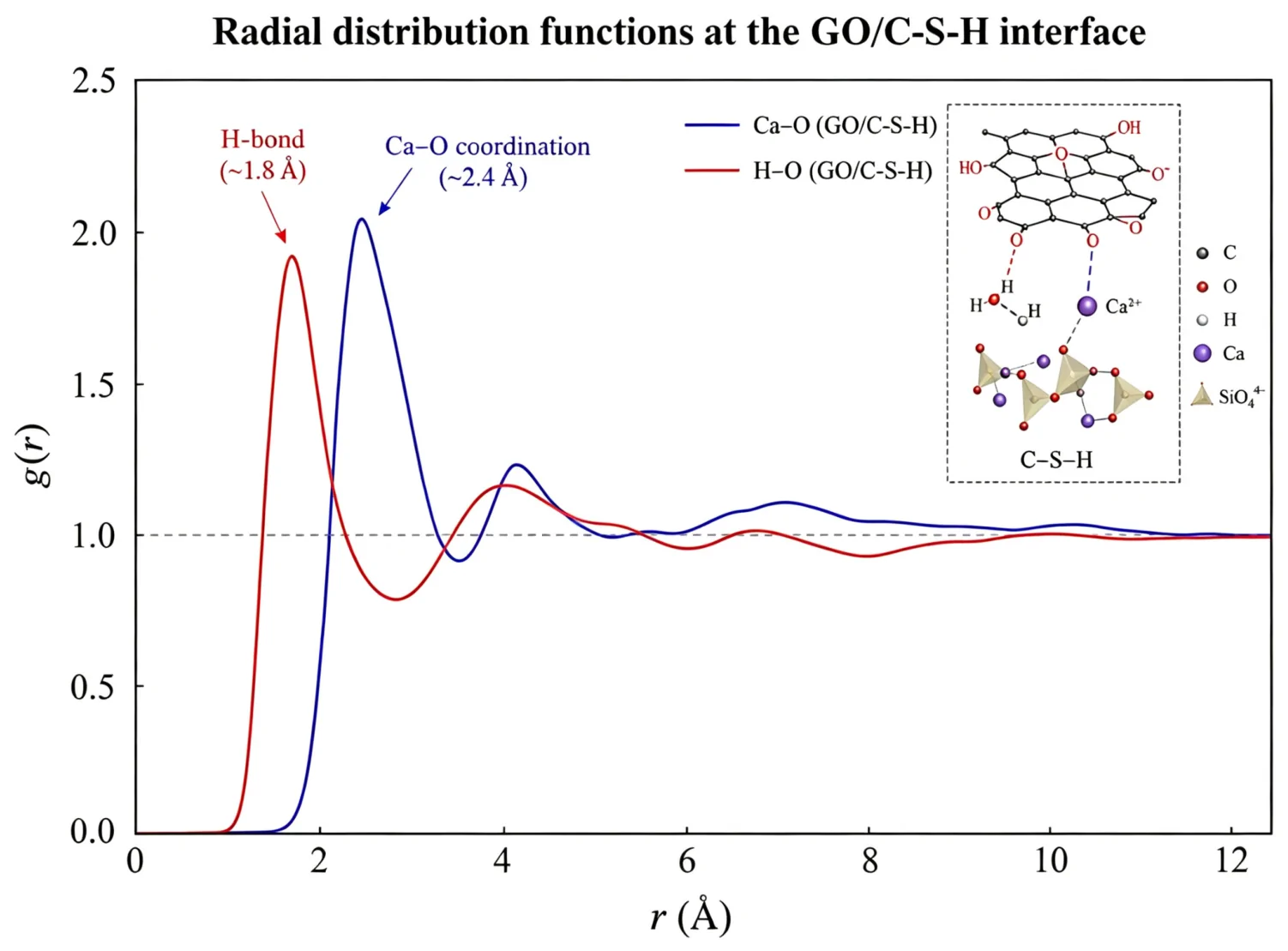
Radial distribution functions between GO oxygen atoms and calcium/hydrogen atoms in the C-S-H matrix.

The RDF analysis provides structural evidence for the short-range interactions at the GO/C-S-H interface. The Ca-O pair exhibits a distinct first peak near 2.4 Å, which is associated with calcium–oxygen coordination between exposed calcium sites in C-S-H and oxygen-containing groups on GO. The H-O pair shows a first peak near 1.8 Å, corresponding to hydrogen bonding between interlayer water or surface hydroxyl groups and GO oxygen atoms. These peak positions and relative intensities are consistent with the RDF profiles calculated from the equilibrium trajectories. The corresponding RDF profiles are shown in Figure 6.

The pronounced first-shell peaks indicate that oxidation introduces additional local ordering at the interface. As the separation distance increases, both RDF curves gradually oscillate around *g*(*r*) = 1, indicating that the short-range order weakens at larger distances. Calcium coordination and hydrogen bonding therefore act as interfacial vibrational bridges, which is consistent with the increase in binding energy and the higher thermal-conductivity retention of oxidized GO.

The combined binding-energy and RDF results indicate that oxidation changes both the magnitude of adhesion and the microscopic coupling pathway. Pristine graphene interacts more weakly with the hydrated substrate, whereas oxygen-containing groups in GO create localized coordination and hydrogen-bonding bridges. Stronger interfacial binding should not be interpreted as a direct increase in the absolute conductivity of GO, because oxidation simultaneously disrupts the sp^2^ carbon network and increases intrinsic phonon scattering. Instead, the strengthened interface helps explain why embedding produces a smaller additional conductivity penalty in GO than in pristine graphene, consistent with the increase in conductivity-retention ratio.

### 3.4. Phonon Density of States and Scattering Mechanism

The PDOS spectra reveal distinct responses of graphene and GO to the C-S-H substrate. For pristine graphene, embedding in C-S-H produces a clear shift of the characteristic high-frequency peak from approximately 58.32 to 53.74 THz, accompanied by a reduction in peak intensity of approximately 32%. The out-of-plane spectrum is also suppressed. These changes indicate substrate-induced phonon softening and a reduction in the number or contribution of vibrational modes that participate effectively in thermal transport.

**Figure 7:**
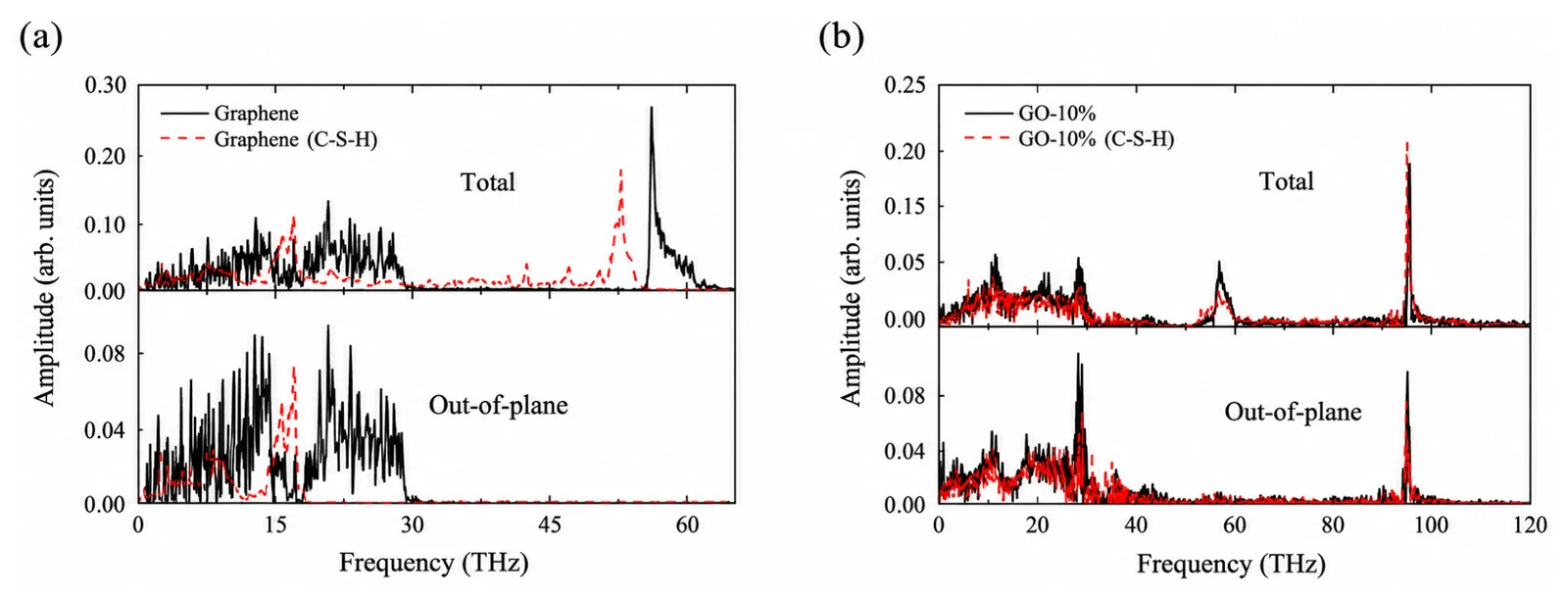
Total and out-of-plane phonon density of states of graphene and GO in isolated and C-S-H-embedded states. Panels: (**a**) total and out-of-plane PDOS of pristine graphene in isolated and C-S-H-embedded states; (**b**) total and out-of-plane PDOS of GO at R = 10% in isolated and C-S-H-embedded states.

The PDOS curves of isolated GO at *R* = 10% and GO embedded in C-S-H are much closer. The characteristic high-frequency peak remains near 97.23 THz, and only limited changes occur in the low-frequency out-of-plane component. The stable spectrum indicates that the vibrational behavior of GO is controlled mainly by its own functional groups and local distortion, while the matrix produces a smaller additional effect. The corresponding total and out-of-plane PDOS spectra are shown in Figure 7.

The thermal-conductivity, heat-flux, binding-energy, RDF, and PDOS results form a consistent mechanism. Pristine graphene provides an ordered and highly conductive transport path, but this path is sensitive to the heterogeneous C-S-H environment. GO has lower intrinsic conductivity because of oxidation-induced disorder, but its interface with C-S-H is stronger and its vibrational spectrum is less sensitive to further disturbance. Therefore, oxidation improves thermal stability in the matrix while reducing the absolute transport capability of the sheet. Selection of a graphene-based filler should consider both quantities rather than intrinsic conductivity alone.

The PDOS response provides the vibrational counterpart to the interfacial and heat-flux observations. The shift and suppression of graphene modes after embedding indicate that the C-S-H environment modifies the frequencies and populations available for heat-carrying phonons, whereas the smaller spectral change in GO suggests that oxidation-induced disorder has already broadened and localized the relevant vibrational states. The combined evidence supports a crossover from substrate-dominated additional scattering in pristine graphene to defect-dominated scattering in oxidized GO.

### 3.5. Implications for Cementitious Nanocomposites

At the macroscopic scale, the thermal performance of a graphene-based cementitious composite depends on filler dosage, dispersion, orientation, sheet contact, pore structure, moisture condition, and the formation of a continuous heat-transfer network. The nanoscale simulations do not directly predict the bulk thermal conductivity of concrete, but they clarify the substrate effect acting on an individual graphene or GO sheet. Graphene offers higher absolute conductivity, whereas GO provides stronger interfacial compatibility and a higher conductivity–retention ratio after embedding. Therefore, filler selection for cementitious materials should consider conductivity retention after interaction with C-S-H together with intrinsic thermal conductivity.

An intermediate oxidation degree may provide a practical balance between intrinsic conductivity, aqueous dispersion, and interfacial stability. Excessive oxidation produces stronger interfacial interaction but substantially lowers the conductivity of the sheet, whereas insufficient oxidation preserves a highly conductive carbon network but results in weaker interaction with C-S-H and greater sensitivity to matrix-induced scattering. The present results support conductivity retention as an additional nanoscale screening metric, but they do not establish a unique optimum oxidation degree or dosage for bulk cementitious composites.

Several limitations should be recognized. First, the absolute conductivity was obtained with a single ReaxFF framework and therefore remains force-field-dependent. Second, the finite model lengths are far smaller than macroscopic graphene dimensions, so the extrapolated infinite-length conductivity is model-dependent. Third, the single-sheet C-S-H model does not explicitly represent agglomeration, pore connectivity, variable moisture, or hydration evolution in real cement paste. Future work should benchmark the thermal response against alternative carbon potentials and/or first-principles phonon data, extend the model length and configuration sampling, and couple atomistic predictions with mesoscale simulations and experimental measurements of cementitious nanocomposites.

## 4. Conclusions

(1) Pristine graphene has the highest intrinsic thermal conductivity, but the value decreases from 1854.6 to 1264.2 W/(m·K) after embedding in C-S-H. The retention ratio of 0.68 demonstrates a strong substrate-induced scattering effect.
(2) Oxidation reduces the absolute conductivity of GO but weakens the additional conductivity loss caused by C-S-H. At *R* = 20%, GO retains more than 90% of its isolated-sheet conductivity, and the retention ratio approaches a plateau when *R* is approximately 10% or higher.
(3) Heat-flux vector analysis shows that C-S-H markedly broadens the transport-direction distribution of graphene, whereas the vector field of GO changes only slightly after embedding. Intrinsic oxidation-induced disorder therefore dominates the scattering behavior of GO.
(4) The binding energy increases with oxidation degree, and the xy-plane of C-S-H exhibits the strongest interaction with GO. RDF first peaks near 2.4 and 1.8 Å indicate Ca-O coordination and hydrogen bonding, respectively, providing structural support for the strengthened GO/C-S-H interface.
(5) PDOS analysis confirms pronounced phonon softening in embedded graphene, while the vibrational spectrum of GO remains comparatively stable. The results reveal a trade-off between intrinsic thermal conductivity and matrix-induced thermal stability and provide a molecular basis for selecting graphene-based fillers in cementitious materials.

The main limitations of the present study are the use of a single ReaxFF parameterization and finite-size RNEMD extrapolation. Future work will focus on cross-potential benchmarking, larger model lengths and independent interfacial configurations, followed by multiscale and experimental validation in bulk cementitious composites.

## Figures and Tables

**Table 1:** Main model and simulation parameters.

Category	Parameter	Value
C-S-H model	Ca/Si ratio	Approximately 1.67
C-S-H model	*Q*^0^/*Q*^1^/*Q*^2^ distribution	11.6%/65.1%/23.3%
C-S-H model	Interlayer water density	Approximately 1.0 g/cm^3^
Graphene/GO	Sheet transverse size	Approximately 2.45 nm
Graphene/GO	Hydroxyl/epoxy ratio	1:1
Graphene/GO	Oxidation degree, *R*	0–20%
Simulation	Force field	ReaxFF
Simulation	Time step	0.25 fs
Equilibration	NPT condition	300 K and 1 atm for 0.5 ns
Equilibration	NVE relaxation	0.1 ns
RNEMD	Energy-exchange interval	Every 1000 time steps
RNEMD	Total simulation time	3.0 ns

## Data Availability

The data supporting the findings of this study are available from the corresponding author upon reasonable request.
